# One-stage scalp reconstruction using single-layer dermal regeneration template and split-thickness skin graft: a case series

**DOI:** 10.1007/s10006-024-01292-5

**Published:** 2024-10-04

**Authors:** Ciro Emiliano Boschetti, Giorgio Lo Giudice, Samuel Staglianò, Annalisa Pollice, David Guida, Roberta Magliulo, Giuseppe Colella, Fabrizio Chirico, Mario Santagata

**Affiliations:** 1 Multidisciplinary Department of Medical-Surgical and Dental Specialties. Oral and Maxillofacial Surgery Unit, University of Campania “Luigi Vanvitelli”, Naples, 80138 Italy; 2 Department of Biomedical, Dental Sciences and Morphological and Functional Images, University of Messina, Via Consolare Valeria 1, Messina, 98124 Italy

**Keywords:** Scalp reconstruction, Cutaneous malignancies, Skin carcinomas, Dermal regeneration template, Head and neck surgery

## Abstract

**Purpose:**

Scalp full-thickness defects reconstruction following the resection of skin carcinoma poses significant challenges due to scalp anatomy complexity and limited vascularity. Despite various techniques available, including tissue expansion and local flaps, no single method stands as the gold standard. Moreover, cases requiring adjuvant radiotherapy further complicate reconstruction, demanding durable solutions. This study explores the efficacy of Integra^®^ Dermal Regeneration Template Single Layer (Integra DRTSL) followed by split-thickness skin grafting (STSG) in one-stage scalp reconstruction post oncologic resection.

**Methods:**

A retrospective analysis was conducted on patients undergoing this procedure from January 2020 to October 2023. Surgical outcomes, including graft take rates, complications, and adjuvant therapy tolerability, were assessed.

**Results:**

Results demonstrated successful reconstruction in the majority of cases, with a complete graft take rate of 77% and minimal complications. Notably, the single-stage approach facilitated timely initiation of adjuvant therapy, crucial for oncologic management. Healing times were notably reduced (< 60 days), enabling early radiotherapy commencement. No local recurrences were observed during the 16-month follow-up.

**Conclusion:**

The use of Integra DRTSL with STSG in one-stage reconstruction presents a promising alternative, offering optimal cosmetic and functional outcomes with low complication rates. This approach streamlines the reconstruction process, ensuring timely adjuvant therapy initiation and maximizing patient outcomes, especially in the context of scalp cutaneous tumors requiring radiotherapy.

**Clinical trial number:**

This research was conducted in accordance with the Declaration of Helsinki and approved by the Ethics Committee of University of Campania “Luigi Vanvitelli” (protocol code N. 0013333, 29 April 2021)

## Introduction

The reconstruction of large full-thickness scalp defects presents significant challenges to surgeons due to the complex anatomy of the scalp, limited mobility, and poor vascularity of the calvaria [1, 2]. Various techniques, including primary closure, tissue expansion, skin grafting, local flaps, and microvascular free tissue transfer, have been employed for scalp defect repair [3, 4]. However, each technique has its limitations and associated complications, and no single best method has been established. Additionally, the requirement for radiotherapy in cases of cutaneous malignant neoplasms further complicates the reconstruction process, necessitating viable and durable reconstruction to withstand the effects of radiotherapy.

In recent years, the use of artificial skin substitutes or acellular dermal matrix followed by skin grafting has emerged as a promising reconstructive approach of different anatomical units [5–7]. Our study specifically investigates the utilization of the Integra^®^ Dermal Regeneration Template Single Layer (Integra DRTSL) (Integra LifeSciences, Princeton, New Jersey, USA), a dermal regeneration template developed in the early 1980s, in reconstructing scalp defects resulting from the resection of skin carcinomas. Our approach involves employing a single-step surgical technique to address the challenges associated with scalp reconstruction after oncologic resection, which often requires immediate reconstruction due to the aggressive nature of scalp malignancies. Traditional techniques like tissue expansion are not feasible in these cases, and alternative approaches such as local flaps, regional tissue transfer, and free tissue transfer have limitations and associated morbidity [8–10]. The aim of this research is to review our institution’s experience with the Integra DRTSL, followed by split-thickness skin grafting (STSG), as a reconstructive strategy for scalp defects. We highlight the advantages of using the Integra template, which allows scalp wounds to be covered even in cases where periosteum or temporalis muscle/fascia are not intact, and the feasibility and costs of the one-stage procedure. By evaluating its efficacy, advantages and limitations, we aim to contribute to the body of knowledge surrounding scalp reconstruction techniques, ultimately improving patient outcomes in the management of scalp cutaneous tumors. Reducing operative times, the number of procedures performed and ensuring maximum safety and effectiveness, thus facilitating shorter recovery times and faster completion of the treatment process.

## Materials and methods

We performed a retrospective review of patients who underwent reconstruction of large scalp defects using the application of the Integra^®^ single-layer wound matrix followed by STSG in a single-stage surgery, from January 1, 2020, to October 31, 2023. All patients were treated at the University of Campania Luigi Vanvitelli and underwent surgical resection of a malignant cutaneous neoplasm of the scalp. A variety of reconstruction options, such as local flaps, healing by secondary intention, or the application of different techniques, were discussed with these patients. Staging was performed through Computed Tomography (CT) scans and ultrasonography of the latero-cervical, occipital and parotid lymph nodes. Written informed consent was obtained from all patients for the proposed surgical procedure. Several of these patients were also referred for adjuvant radiotherapy based on the recommendations of the multidisciplinary cutaneous tumor board at the University of Campania Luigi Vanvitelli. This study was reviewed and approved by the ethics committee of the University of Campania Luigi Vanvitelli [prot. 0013333, 29 April 2021].

### Patients characteristics

A total of *n* = 18 patients, with a median age of 73,8 years (range 55–89) were included (Table 1). All resected scalp lesions were cutaneous malignant tumors. Histological types were squamous cell carcinoma (8 patients), malignant melanoma (4 patients), basal cell carcinoma (3 patients), merkel cell carcinoma (2 patients) sarcoma (1 patients). Twelve patients received adjuvant therapy consisting in radiotherapy (*n* = 9) or immunotherapy (*n* = 3). The mean surface of the scalp wound was 45 (cm2) (range 13–97). The average total time of surgical procedures was 94 min (60–127 min).

**Table 1:** Patient characteristics

Patient Sex/Age-years	Defect Size mm	Histological findings
F/73	43 × 31	Malignant melanoma
M/84	73 × 61	Basal cell carcinoma
M/89	45 × 38	Sarcoma
M/76	98 × 76	Malignant melanoma
M/60	80 × 67	Squamous cell carcinoma
M/59	120 × 78	Squamous cell carcinoma
M/69	56 × 80	Merkel cell carcinoma
M/71	80 × 59	Squamous cell carcinoma
M/65	92 × 97	Squamous cell carcinoma
M/79	55 × 63	Malignant melanoma
M/88	107 × 84	Squamous cell carcinoma
F/64	89 × 72	Basal cell carcinoma
M/84	132 × 94	Squamous cell carcinoma
F/73	65 × 79	Squamous cell carcinoma
M/87	94 × 45	Basal cell carcinoma
M/72	36 × 52	Merkel cell carcinoma
F/81	102 × 94	Squamous cell carcinoma
F/55	38 × 43	Malignant melanoma

### Surgical procedure

**Fig. 1:**
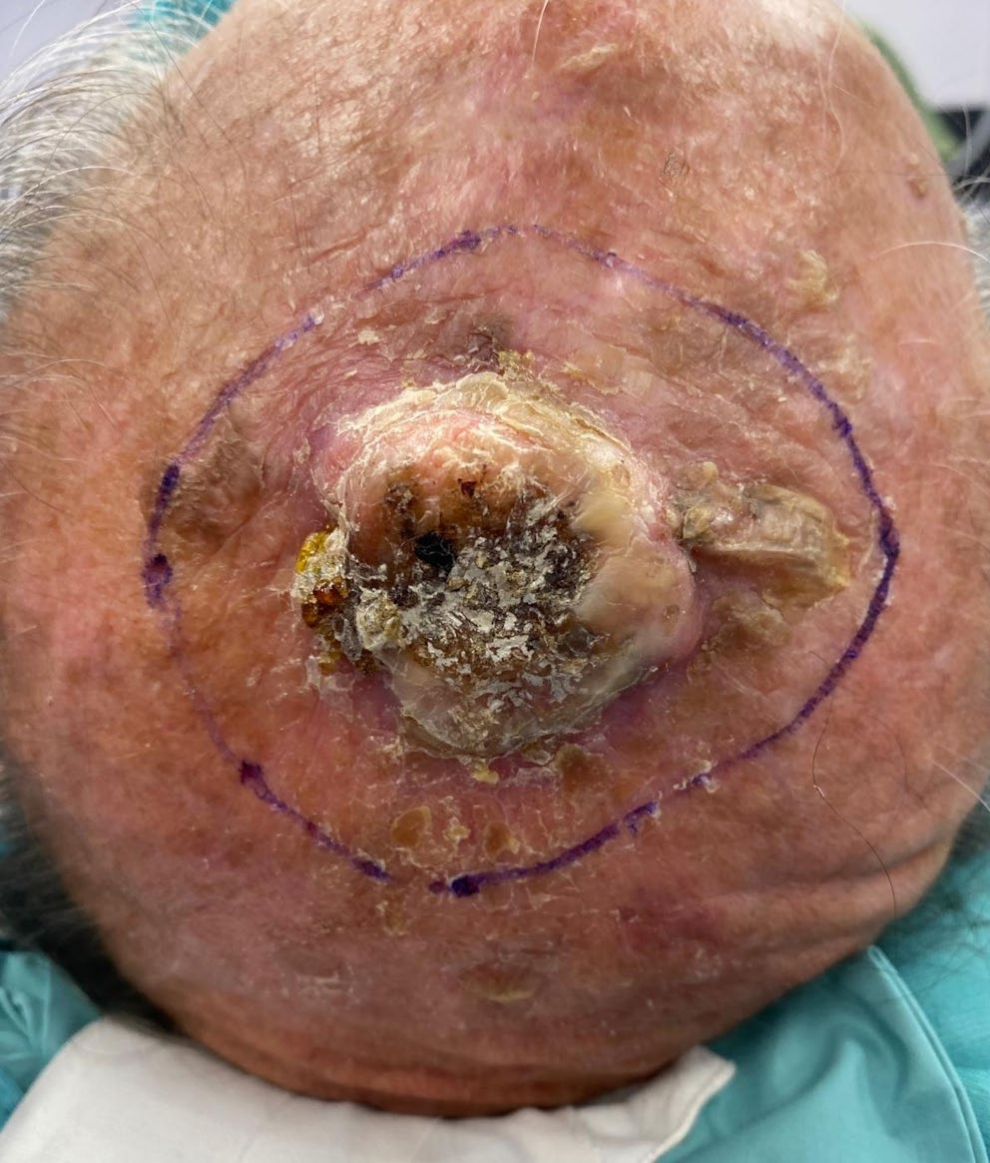
Invasive squamous cell carcinoma of the scalp

**Fig. 2:**
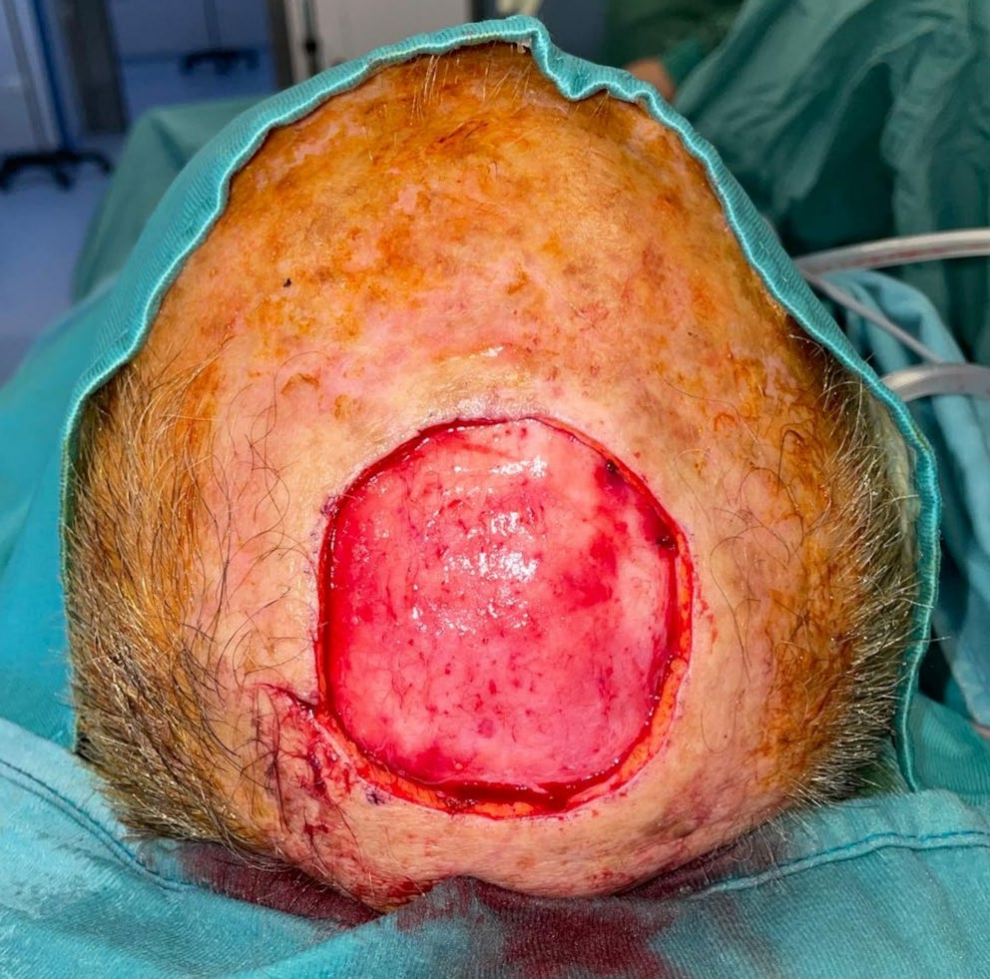
Tumor resection with removal of the periosteum underlying the calvaria

Under general anesthesia, the primary scalp lesion was surgically excised from all patients including employing frozen sections to control the margins. The margins of excision were evaluated according to the tumor histotype, as well as the preoperative characteristics of the lesion, assessed by US and CT imaging. In squamous cell histotype tumors, 62% required removal of the lesion to reach the pericranium, 38% had to have the pericranium and the bony cortex of the scalp removed. As for the excision margins, in all patients with squamous carcinoma, a free margin of > 10 mm was maintained in relation to the size of the lesion. Patients with basal cell histotype and Merkel cell carcinoma, required a deep removal up to the pericranium. For the first, a peri-lesion removal was chosen for the excision margins, and for the second, a free margin > 20 mm. In the treatment of melanoma lesions, the Breslow index was taken into account for the evaluation of margins. If removal of the periosteum overlying the calvaria was planned, a pericranium flap was performed. When needed, the bone was removed by use of piezoelectric instrumentation until macroscopically healthy, bleeding bone tissue was reached. A purse-string suture was used around the outside edge of the scalp defect in certain cases to slightly reduce the wound bed. Prior to reconstruction, all patients were deemed to have clear margins. Before reconstruction, the wound bed was thoroughly irrigated with sterile saline solution and hemostasis was achieved using bipolar cautery (Figs. 1 and 2).

**Fig. 3:**
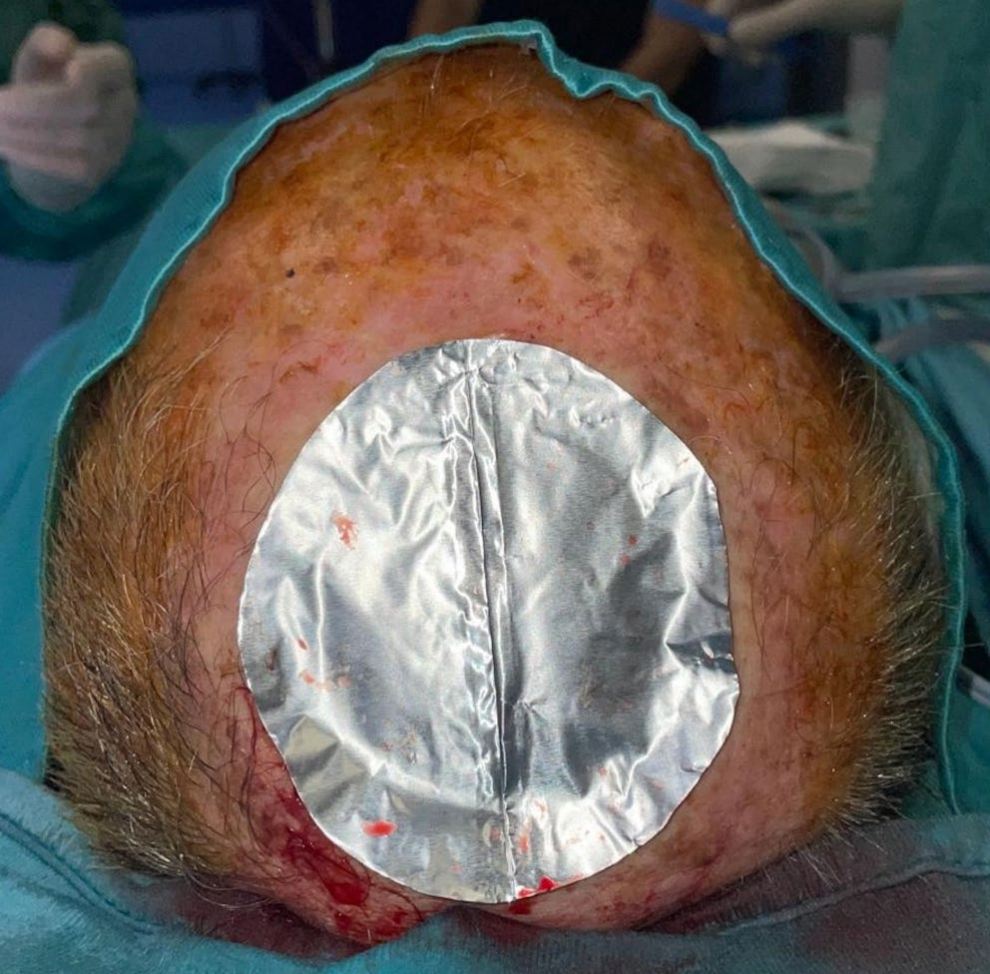
A polyurethane template was used to guide the cutting of the Integra DRTSL to the desired size

**Fig. 4:**
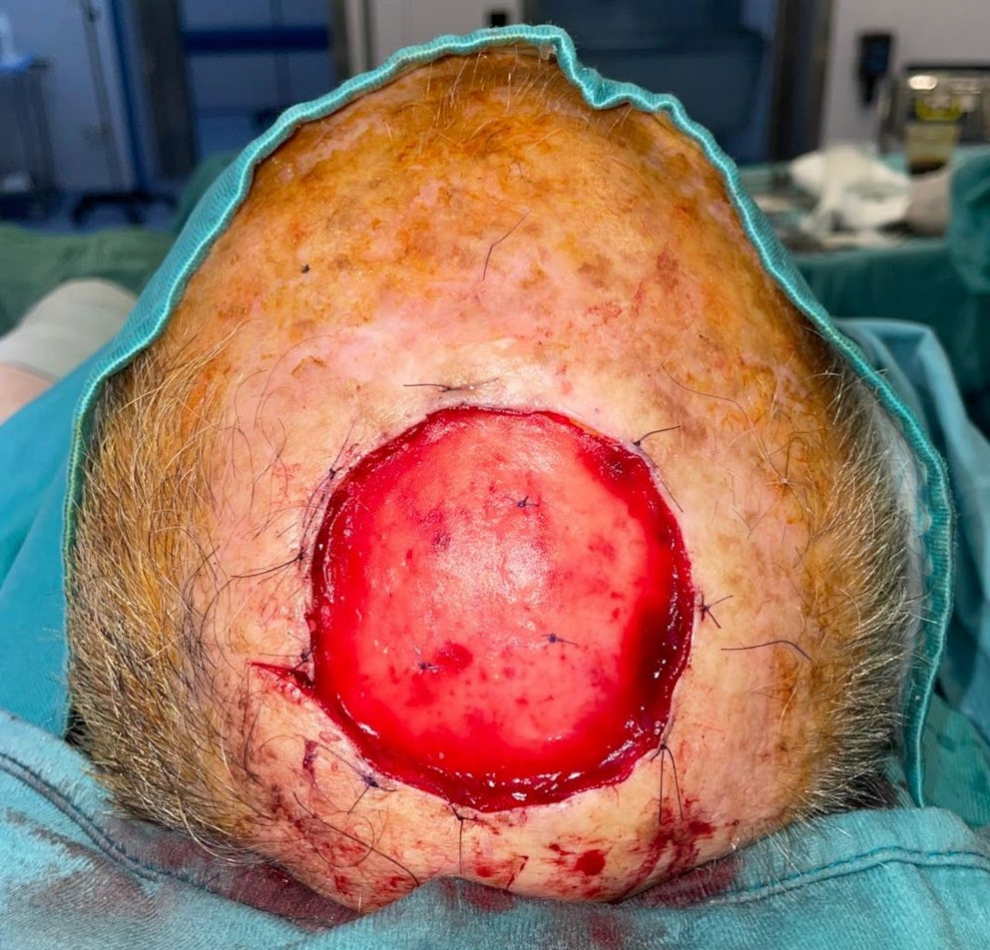
Placement of Integra DRTSL in the wound bed and its fixation by Vicryl sutures

Following the manufacturer’s instructions for proper material preparation, a polyurethane template was used to guide the cutting of the Integra DRTSL to the desired size. The Integra DRTSL was then placed in the wound bed and sutured by Vicryl sutures (Figs. 3 and 4).

**Fig. 5:**
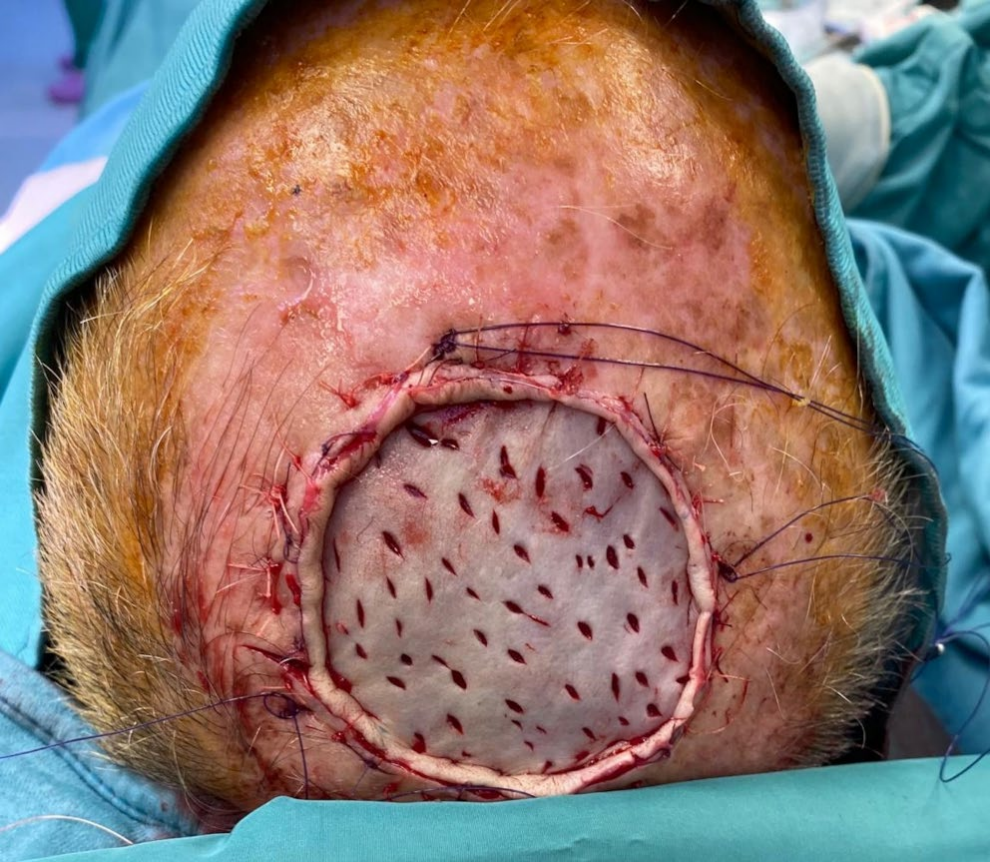
The STSG is placed and secured to the wound bed by rapid Vicryl sutures

After irrigating the surface of Integra DRTSL with gentamicin, the split-thickness skin graft is placed and secured to the wound bed by rapid Vicryl sutures, thus concluding the single-step procedure (Fig. 5).

After applying moist gauze with hyaluronic acid to the graft, it was secured with two dressings (Polyurethane dressing / hydrophilic sponges) soaked in gentamicin sulfate solution. The dressing had a nonadherent contact layer, a foam core, and an outer polyurethane layer to protect the wound during postoperative care. To cover and protect the entire wound, the second layer of dressing was cut slightly larger than the wound bed. The nonadherent layer allowed for easy removal while causing minimal disruption to the tissue beneath, while the sponge core allowed the dressing to be flexible and absorbent. Vicryl sutures and skin staples were used to secure each layer of the dressing to the surrounding normal skin at the defect’s edge.

### Post operative care and follow-up

All patients were seen in the clinic for a wound check at 1 week after the procedure. The dressings were removed, the wound edges were debrided gently as needed to remove any crusting that had formed, and the moist gauze with hyaluronic acid was replaced. After two weeks, the patient is instructed to keep the surgical site open and apply gentamicin ointment.

## Results

Regarding the 18 patients who benefited of the reconstruction using our technique, 100% got graft engraftment, 14 (77%) had a 100% graft take rate without any complication. Four patients (23%) had postoperative complications that resulted in a delayed healing period. One of them suffered partial skin graft loss with a 60-day healing delay. There were no major complications that required additional surgery (Table 2).

**Table 2:** Patient results

Patient	Postoperative Radiotherapy	Length of Follow-up /months	Skin Graft Performed Take, %	Wound healing/days	Complications
1	No	12	100	36	No
2	No	18	100	34	No
3	No	22	100	54	No
4	No	20	100	32	No
5	Yes	26	100	44	No
6	Yes	12	85	82	Post-actinic erythema Partial skin graft loss
7	No	26	100	32	No
8	Yes	8	100	46	Post-actinic erythema
9	Yes	11	85	79	Partial skin graft loss
10	No	28	100	43	No
11	Yes	9	100	36	Post-actinic erythema
12	Yes	21	100	50	G2 epidermyolisis after RT
13	Yes	23	75	120	Partial skin graft loss
14	No	9	100	31	NO
15	Yes	13	100	48	Post-actinic erythema
16	No	22	100	39	No
17	Yes	2	90	88	Partial skin graft loss
18	No	7	100	57	No

Postoperative radiotherapy was recommended in 9 of 18 patients (50%). All patients underwent a protocol with moderately hypofractionated, intensity-modulated radiotherapy (IMRT). A total dose of 55 Gy was given in 20 fractions of 2,75 Gy in a 20-day period. Six patients (66%) did not show any breakdown of the graft after IMRT and tolerated the treatment without any major toxic effects. Post-actinic erythema was evident in 30% of cases, and only 5% of cases showed the presence of grade G1-G2 epidermyolysis. Radiation therapy was started between 45 and 60 days after surgical treatment and immunotherapy treatment between 30 and 45 days after tumor excision.

**Fig. 6:**
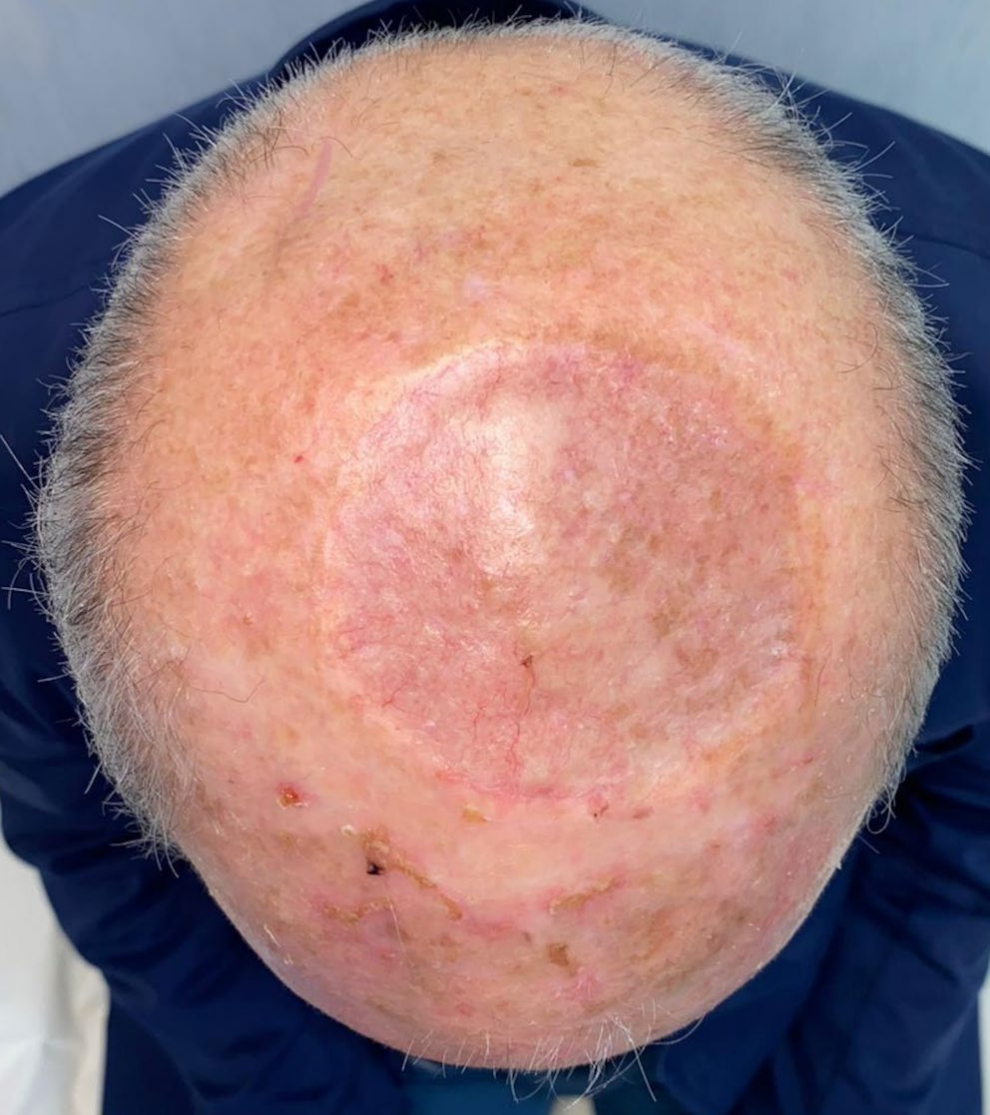
Clinical follow-up 12 months after surgery

**Fig. 7:**
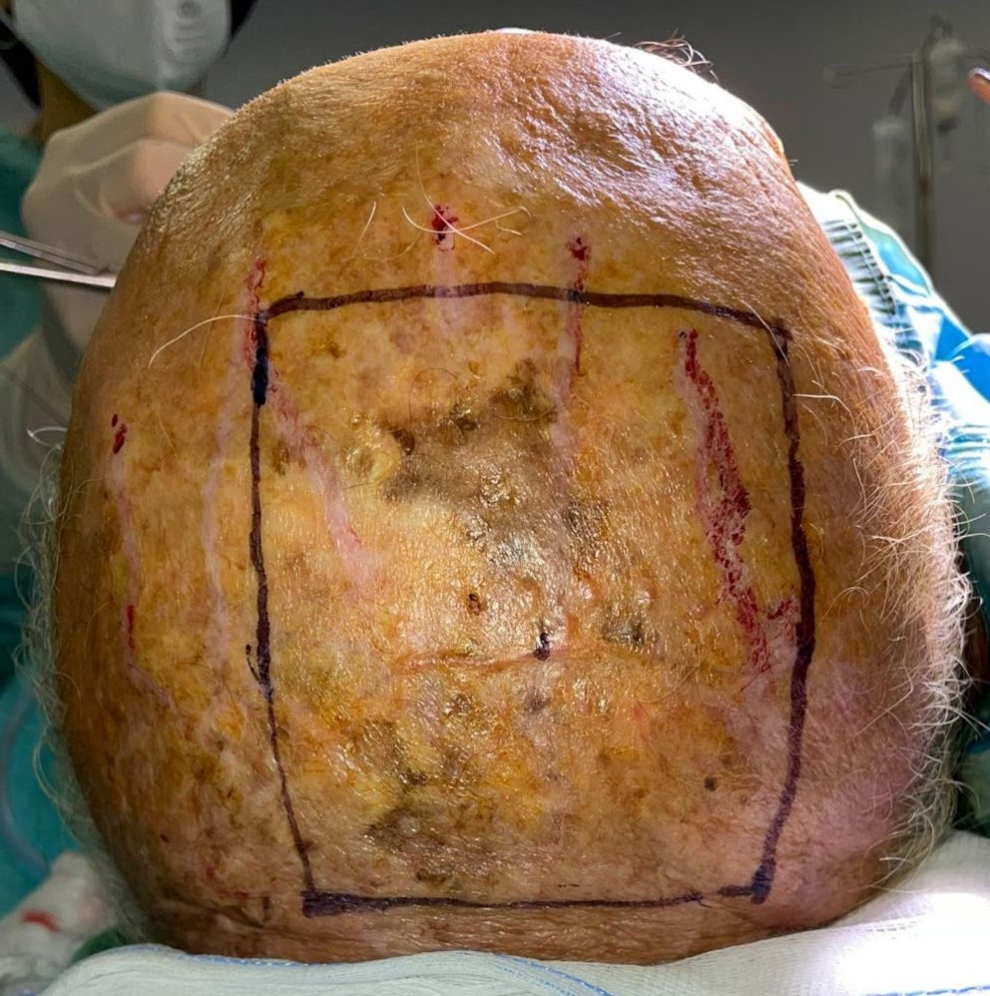
Malignant melanoma of the scalp

**Fig. 8:**
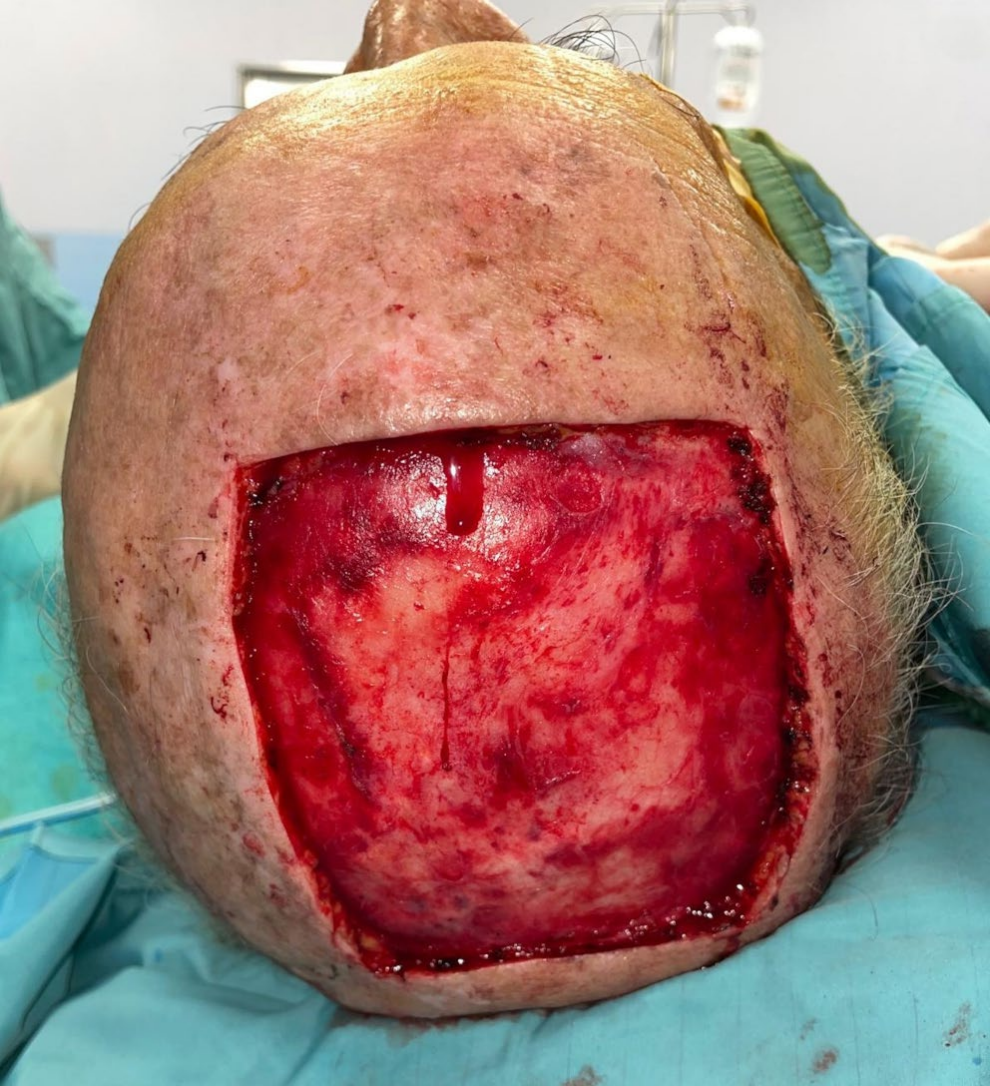
Resection of the tumor with a scalp wound surface of about 91 cm^2^

**Fig. 9:**
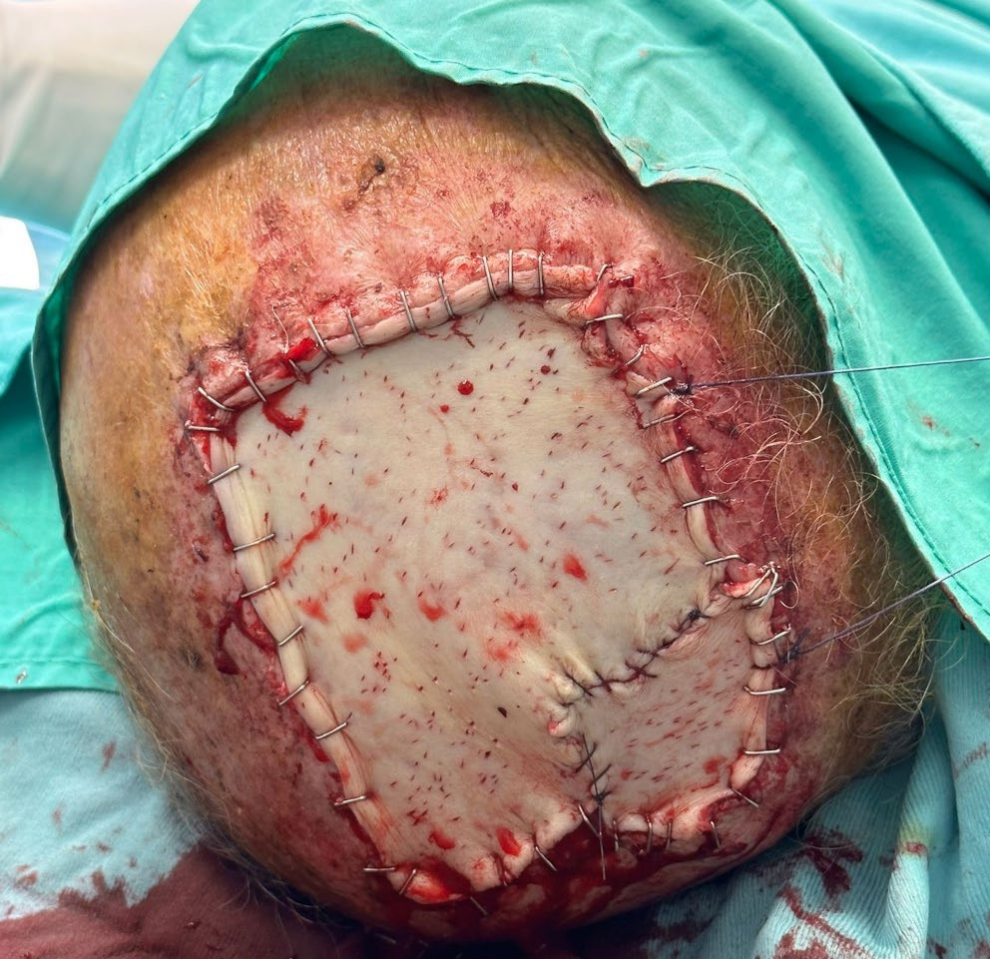
One-stage reconstruction with Integra DRTSL and overlying STSG sutured at the wound edges using skin staples and Vicryl sutures

**Fig. 10:**
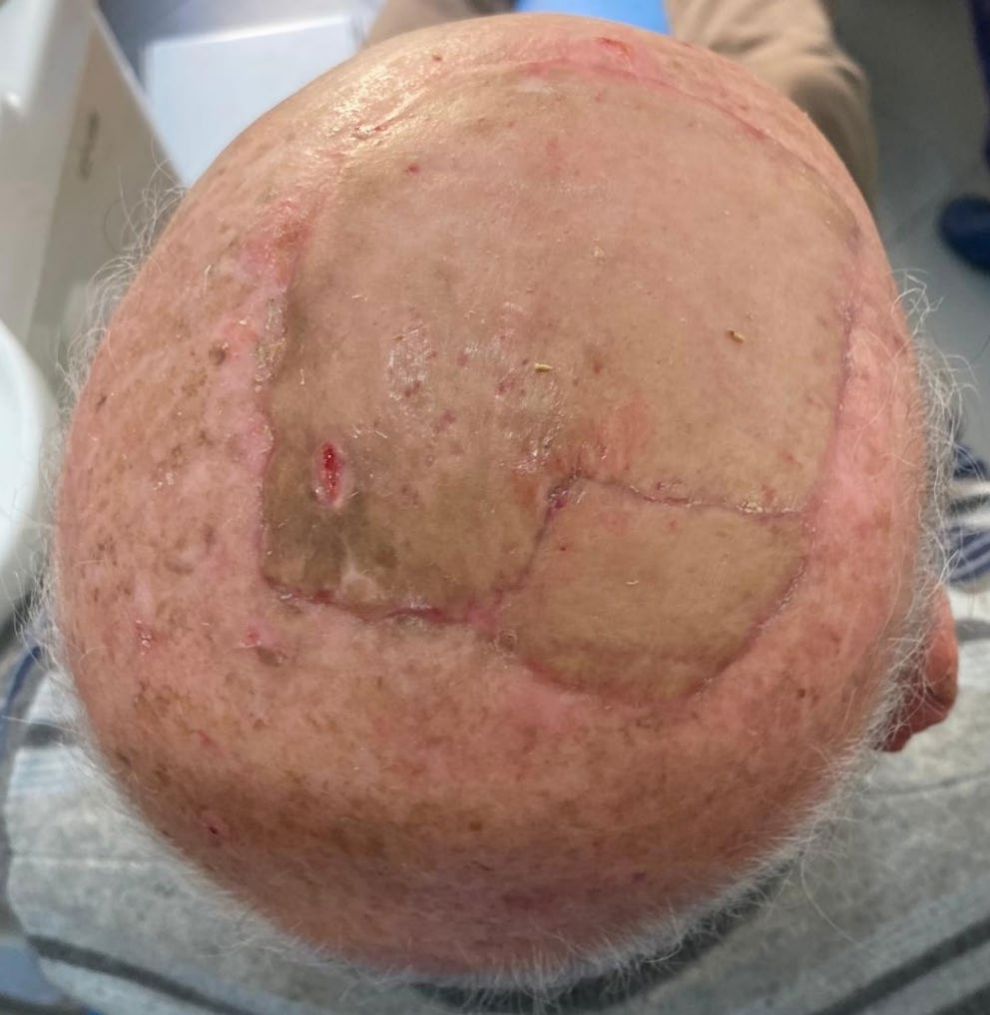
Clinical follow-up 6 months after surgery

The median healing time between cancer resection and the end of dressing care was 40 days in the 14 patients (77%) who did not have any postoperative complications, achieving an excellent aesthetic result (Fig. 6), while the mean healing time in the entire cohort was 53 days. The average duration of follow-up was 16 months (range: 2–28 months). There was no local recurrence during this period, but four patients treated for scalp melanoma (*n* = 1) (Figs. 7-10), squamous cell carcinoma (*n* = 2), and Merkel cell carcinoma (*n* = 1) experienced metastatic relapses (cervical and parotid nodes for the squamous cell carcinoma, hepatic metastatic tumor for the Merkel cell carcinoma, and multiples localization for the melanoma).

## Discussion

The scalp defects reconstruction after tumor ablation still represents a challenge for the surgeon. The struggle is due to the scalp intrinsic anatomical features, such as lack of redundant tissue, poor vascularization, limited mobility, and due to the patients’ characteristics. As a matter of facts, the patients who undergo this type of surgery are usually old and affected by multiple comorbidities [11, 12]. Furthermore, they most frequently present large tumors that require extensive resection to achieve surgical radicality and often need adjuvant radiotherapy. Through this retrospective study, we aim to provide insights into the use of the Integra DRTSL as a viable reconstructive strategy for such complex scalp defects. Different techniques are in use for large full–thickness scalp defects: primary intention healing, advancement, rotation or transposition flaps, loco-regional and free-flaps, STSGs or full-thickness skin graft (FTSG) [1]. During recent years, the use of the Integra DRT has become more and more popular as a valid alternative for scalp defects repair, especially in bald patients. This artificial dermal substitute is characterized by two layers: an outer silicone layer, that works as a protective layer, and an inner cross-linked matrix layer, that acts as scaffold for fibroblasts, macrophages, lymphocytes, and endothelial cells to migrate from the surrounding tissue to the artificial dermis to finally replace it [13]. The matrix closely resembles dermis in structure and composition, inducing the synthesis of a neodermis and promoting wound healing. Additionally, the Integra matrix can vascularize from the periphery, enabling skin grafting on previously avascular wound beds, including bone and tendon [14]. The reconstructive surgery usually requires two surgical sessions. During the first step surgery, the Integra DRT is placed over the scalp defect immediately after the tumor ablation, after three weeks, a second stage surgery is performed to remove the protective silicone layer and to cover the acellular dermal matrix with a STSG. This allows to avoid the divot, thus the skin contour distortion related to the skin graft alone procedure, and it allows the formation of a robust vascular tissue closure, that ensures a major cranial protection to withstand radiotherapy, if needed [15]. The Integra DRT has been demonstrated to be a reasonable reconstructive option, in terms of aesthetic and functional results in the head and neck district [16–19]. Nevertheless, two surgical times are required and the healing time is quite long (> 50 days up to 3.8 months), especially if radiotherapy is needed. The re–epithelization by secondary intention after DRT alone positioning was also attempted, however the total healing time overcame the 60 days [20]. These features provided the rationale of our study, that attempts to evaluate the feasibility of a single stage reconstructive surgery in which the Integra DRT Single Layer is used. This acellular dermal replacement template consists of a porous three-dimensional matrix comprised of bovine tendon collagen and chondroitin-6-sulfate and it lacks the silicone outer layer. In our study, 18 patients affected by scalp skin cancer underwent reconstruction of the scalp defect by the application of the Integra DRTSL immediately followed by a meshed STSG in a single stage surgery. The procedure was performed also in those cases with exposed bone, with the aid of an underlying pericranium flap. The grafting was successful in most of the patients: the resultant soft-tissue reconstruction was pliable, uniform in texture and color, contour distortion was not observed. Only one patient underwent partial skin graft loss, but we were able to treat him as outpatient without the need to perform a second surgery. In comparison to the commonly practiced algorithms, we observed a reduction in the healing time (< 60 days), thereby allowing the patient to immediately start the radiotherapy when needed. This aspect was of considerable importance especially during the COVID-19 pandemic, when a diagnostic delay was observed in oncological patients [21]. The patients included in our study began the adjuvant treatments within 60 days post-operative. The set-up of a consistent defect replacement avoided significant radiotherapy – related complication in most of them. The safety of the surgical margins was checked by frozen sections and none of the patients presented local recurrences during the 16 months follow-up. According to our study, the use of Integra DRTSL and a STSG in a single step reconstructive surgery can be a viable alternative to the traditional methods of wound closure, in terms of cosmetic and functional results. Unlike the classic two-step treatment, Integra DRTSL guarantees a peripheral neo-vascularization, such as to ensure graft engraftment in a single step. This technical factor, in particular the reduction of waiting times and healing times of the surgical site are the basis for the treatment in single step. Low complication rates, single step surgery, optimal cosmetic outcome, feasibility of the adjuvant radiotherapy due to consistent calvarial protection demonstrated that the procedure can be safely performed as a single–stage approach.

## Data Availability

No datasets were generated or analysed during the current study.
